# Fibre-volume-fraction measurement of carbon fibre reinforced thermoplastic composites using thermogravimetric analysis

**DOI:** 10.1016/j.heliyon.2019.e01132

**Published:** 2019-01-16

**Authors:** M.A.A. Mohsin, L. Iannucci, E.S. Greenhalgh

**Affiliations:** Imperial College London, Faculty of Engineering, Department of Aeronautics, Exhibition Road, London, SW7 2AZ, UK

**Keywords:** Materials science, Physics methods, Materials chemistry

## Abstract

The fibre-volume-fraction (FVF) measurement of fibre-reinforced polymers (FRPs) is crucial in understanding and characterising their mechanical performance. To date, there has not been a standardised, labour-efficient method in determining the FVF of a non-crimp fabric (NCF) carbon reinforced thermoplastic composites (CFRTPs). An alternative method such as thermogravimetric analysis (TGA) has merely been commonly used for carbon-fibre reinforced thermosets (CFRTSs) and glass-fibre reinforced thermosets (GFRTSs). Therefore, this paper reports a range of macro TGA measurements of the constituent materials of two NCF CFRTPs; (i) T700 carbon/polyamide6.6 (PA6.6) and (ii) T700 carbon/polyphenylene sulphide (PPS). The TGA measurements were performed using two different purge gases (air and nitrogen) and the mass degradation with respect to time, temperature and atmospheres were recorded and discussed. Additionally, fractographic analysis on the fibres was carried out to scrutinise and further discuss the findings following the TGA. It was concluded that TGA provided a suitable and reliable alternative method to measure the FVF of CFRTPs.

## 1 Introduction

The fibre-volume-fraction (FVF) of fibre reinforce plastics (FRPs) plays an important role in determining the mechanical performance of a composite material system. The typical method used to determine the FVF comprises the removal of polymer by burn off or acid digestion. Nonetheless, these techniques are labour intensive and require disposal of toxic waste. Therefore, thermogravimetric analysis (TGA) has been identified as an alternative as method [1, 2].

The standardised national and international TGA procedures described [3, 4, 5, 6] were established from glass fibre reinforced polymers (GFRPs) whereas the use of TGA in measuring the FVF is not standardised for CFPRPs. The process of TGA measurements of the constituents of the CFRTP used in this research are described in the following section. This procedure was conducted to measure the degradation (in percentage of original mass) with respect to temperature, time and atmosphere. The mass reduction occurs following sublimation, evaporation, decomposition, chemical reaction, magnetic transformation or electrical transformation.

## 2 Background

Naito and Oguma [7] have shown that TGA can be used to measure a more complex carbon/glass hybrid thermoplastic composites known as ‘CABKOMA’. The samples were 5mm long hybrid rods. Nitrogen was used as the purge gas and the TGA was performed at 30−1000°C at heating rate of 10°C/min. The hybrid thermoplastic composites comprised of unidirectional PAN-based carbon fibre, braids of E-glass fibres and thermoplastic epoxy matrix. TGA was implemented and the weight fractions of the constituent materials: glass fibre, carbon fibre and matrix as well as void have successfully been measured (with coefficient of variation, CV ranging from 1.2−2.8%).

Meier et al. [8] have successfully utilised TGA based procedure to measure the FVF and content of CFRTS composite system consisting T300 carbon twill weave fabrics and EPIKOTE Resin MGS® RIMR 135. The sample size weighed approximately 25mg. Heat was applied at the rate of 20°C/min under nitrogen atmosphere. The procedure was done following Yee and Stephens's technique [9] where the duration of the TGA process is approximately 80 minutes with a maximum temperature of 600°C. This FVF measurement of the CFRTS obtained using the TGA method was also benchmarked against the traditional acid digestion method. It was found that the TGA based methodology yielded about 1.5% higher than the standard acid digestion methodology.

Zhu et al. [10] have reported success in utilising TGA to gauge the changes in volume fraction of carbon fillers in a hybrid carbon nanotube thermoplastic composites. This was achieved by heating the nylon 6 hybrid multiwalled carbon nanotube with carbon fibre fillers up to 800°C at a rate of 20°C/min in nitrogen.

Although there are few research on measuring the FVF of CFRTPs, most of them were done on CFRTS composites and to the author's best knowledge, none of them were based on NCF CFRTP with a different stitching material such as Kevlar®. Hence, the TGA experimental procedure for FVF measurement and fractographic analysis of the NCF CFRTP systems with Kevlar® stitching are inherently novel.

## 3 Materials

The raw materials i.e. both carbon/thermoplastic pre-impregnated fabrics were provided by the TFP Global [11]. The prepregs are NCF biaxial 0/90 T700 carbon pre-impregnated with thermoplastic veils, polyamide 6.6 (PA6.6) and polyphenylene sulphide (PPS), respectively. According to the material supplier, both prepregs should have FVF of approximately 55%.

The CFRTP panels were prepared using hand lay-up method and processed using thermoforming method using a 40-tonne laboratory hydraulic press made by Höfer® following the manufacturer's recommendation. The processing temperature of the T700/PA6.6 and T700/PPS are 275°C and 315°C, respectively. The dwell time for both material systems is approximately 10 minutes.

## 4 Methods

**Fig. 1:**
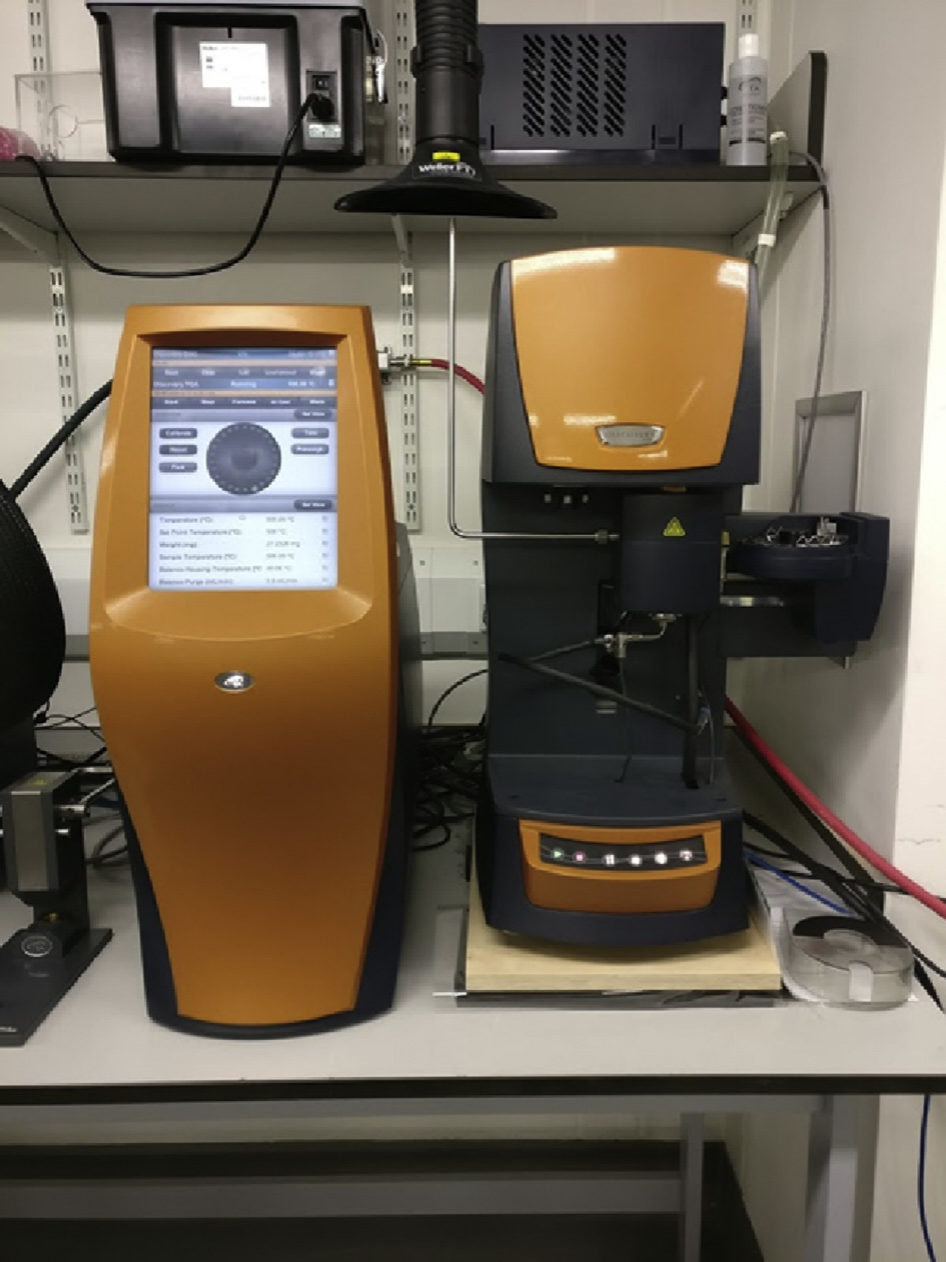
TA Instruments thermogravimetric analyser.

**Fig. 2:**
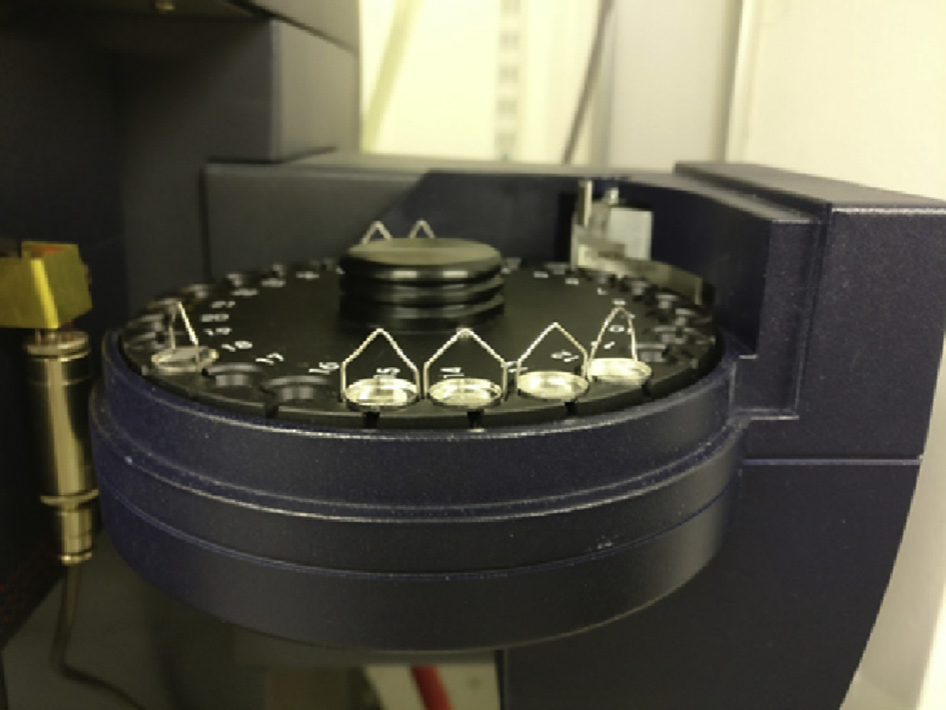
TGA trays.

The test samples (Table 1), obtained from the manufactured panels, were kept in an oven at 40°C for two to three days before performing the TGA, this step allowed to remove, if not minimise the moisture content in the samples. A pycnometer was used to measure the density of the T700/PA6.6 and T700/PPS (Table 2). The average sample mass for the T700/P6.6 and T700/PPS composite is 40mg and approximately 1−1.8mg for each constituent; T700, PA6.6, PPS and Kevlar® (Table 1). The composite samples were taken from different areas across a range of manufactured panels. The constituent materials were taken out of the unprocessed pre-impregnated T700/PA6.6 and T700/PPS, respectively. Subsequently, these samples were prepared for each test and a TA Instruments thermogravimetric analyser was used to record their masses (Fig. 1). The samples were later placed carefully on the TGA trays (Fig. 2). The details of the samples of raw materials such as number of samples used, and average mass are described in Table 2.

**Table 1:** Detail of raw material samples.

Composite material	Constituent materials	Supplier
T700/PA6.6	Fibre: T700	TORAYCA® [12]
	Matrix: PA6.6 (veils)	-
	Stitching: PA6.6	-
T700/PPS	Fibre: T700	TORAYCA® [12]
	Matrix: PPS (veils)	-
	Stitching: Kevlar®	DuPont [13]

**Table 2:** Density of T700/PA6.6 and T700/PPS composite samples measured using a pycnometer after being stored in an oven at 40 °C for three days.

Material	Density, ρ($\mathrm{kg}/m^{3}$)
PA6.6(1)	1.488
PA6.6(2)	1.481
PA6.6(3)	1.485
PA6.6(4)	1.484
PA6.6(5)	1.487
**Average (%)**	1.485
**CV (%)**	0.2
PPS(1)	1.552
PPS(2)	1.553
PPS(3)	1.553
PPS(4)	1.554
PPS(5)	1.551
**Average (%)**	1.553
**CV (%)**	0.1

## 5 Results and discussion

The rate of mass reduction and its mechanism are influenced by the atmosphere present inside the measuring chamber (i.e. purge gas). The thermo-oxidative decomposition occurred due to the presence of oxygen. The pyrolysis (pure thermal decomposition) can be achieved by using inert purge gases such as nitrogen, helium and argon. However, at the time of the TGA test, only nitrogen was available, therefore, this gas was the one used during the procedure.

**Fig. 3:**
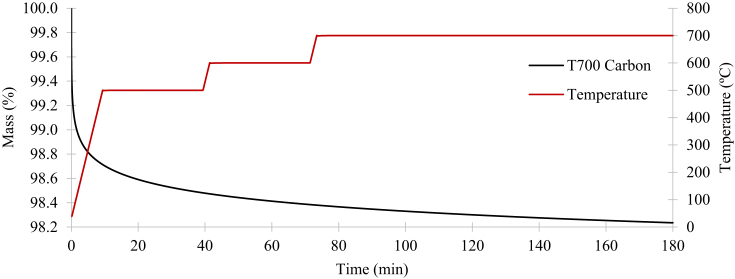
Mass reduction of T700 carbon in nitrogen.

It is critical to demonstrate that the mass of the T700 fibres suffers negligible mass loss under TGA temperatures of up to 700°C (Fig. 3). This was due to the decomposition temperatures of the rest of the polymer (PA6.6 and PPS), as well as the stitching (PA6.6 and Kevlar®) for both CFRTP systems not exceeding 700°C.

The sample mass reduction of T700 carbon fibres during macro TGAs using nitrogen as the purge gas is displayed in Fig. 3. Initially, the temperature was set to ramp at 50°C/min to 500°C. Then, it was set to isothermal for 30 minutes. The same steps were followed to achieve 600°C and 700°C. However, the TGA process was set to be isothermal after reaching 700°C, so that it ran for three hours. Ultimately, the mass percentage of the T700 carbon stays constant at 98.2%. The negligible mass loss of 1.8% could have accounted for dirt and moisture attached to the fibres. Hence, the T700 carbon is virtually unaffected at 700°C.

**Fig. 4:**
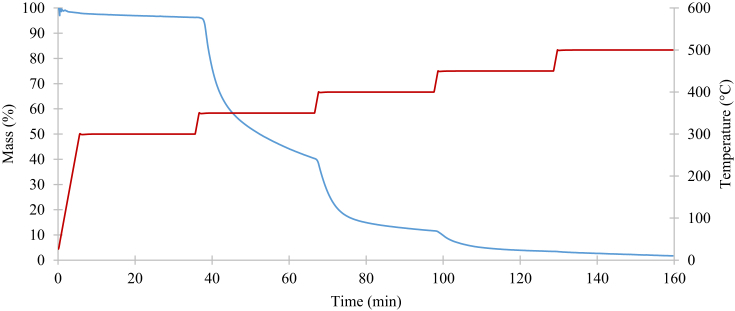
Mass reduction of PA6.6 in nitrogen.

**Fig. 5:**
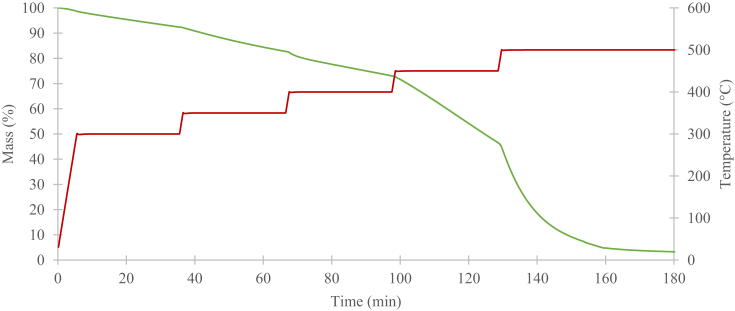
Mass reduction of PPS in nitrogen.

The pyrolysis processes of neat PA6.6 and PPS are depicted in Figs. 4 and 5, respectively. Originally, the temperature was set to rise at 50°C/min to 300°C and then programmed to be isothermal for 30 minutes. The procedure was then repeated at 350, 400, 450 and 500°C. At about 450°C the mass reduction rate of PA6.6 started to become constant and eventually reduced to 0. At this point, it can be confirmed that pyrolysis has occurred. The same was achieved with PPS at 500°C.

**Fig. 6:**
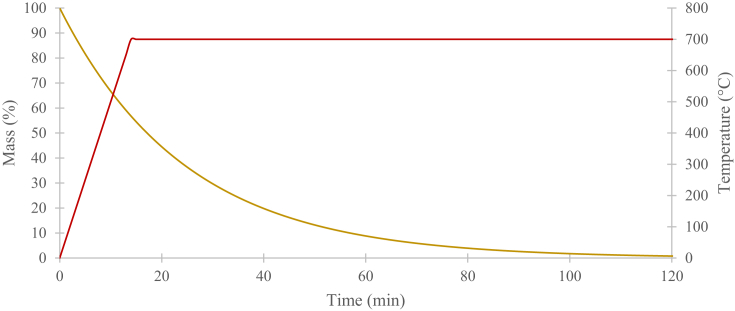
Mass reduction of Kevlar® in nitrogen.

The pyrolysis process of neat Kevlar® fibres is shown in Fig. 6. It has been reported that, depending on the specific Kevlar® aramid fibre type, the decomposition temperature of Kevlar® would be above 500°C [13, 14]. The manufacturer of the T700/PPS prepregs did not specify the exact Kevlar® type used to stitch the NCF, therefore, the temperature was set to ramp at 50°C/min to 700°C and then set to isothermal for two hours, instead of trying to identify the exact decomposition temperature. This procedure provided the means to confirm that the Kevlar® fibres would completely decompose at 700°C.

**Fig. 7:**
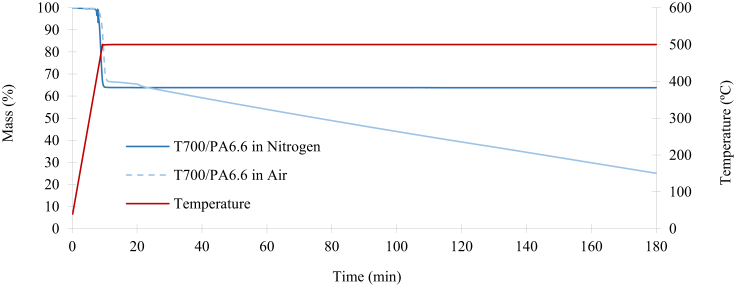
Mass reduction of the T700/PA6.6 in nitrogen and air.

**Fig. 8:**
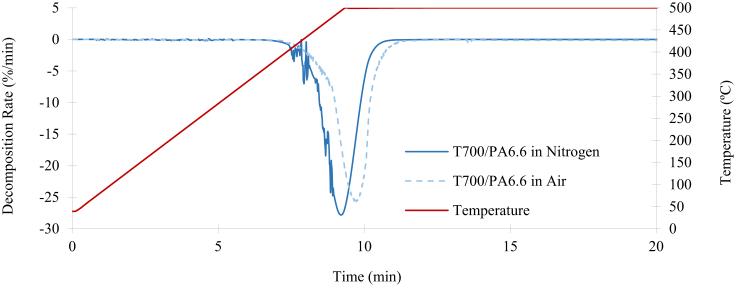
Decomposition rate of T700/PA6.6 in nitrogen and air.

The TGA of T700/PA6.6 in nitrogen and air is illustrated in Fig. 7. Oxidation was detected following a brief exposure to the temperature of 500°C in air. This process led to the mass decomposition of the entire system (denoted by the dashed blue line in Fig. 7). However, the mass reduction did not stabilise even after three hours, which indicated that the fibres were also being oxidised (this will later be confirmed by the SEM micrographs of the fibres following the oxidation process). On the other hand, when nitrogen was used as the purge gas following the same procedure, the mass decomposition stabilises as soon as the temperature reaches 500°C and the mass reduction converges to 63.8% (which indicates the mass percentage of the remaining T700 fibres). The samples' mass degradation rates are shown in Fig. 8. During the three hours of the testing period, the peak degradation rate of the T700/PA6.6 is −27.5%/min in nitrogen and −25.7%/min in air.

**Fig. 9:**
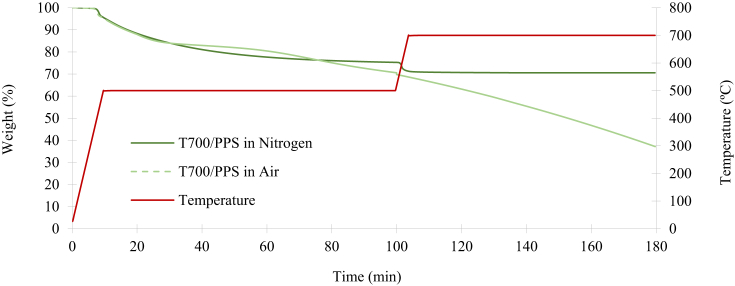
Mass reduction of the T700/PPS in nitrogen and air.

**Fig. 10:**
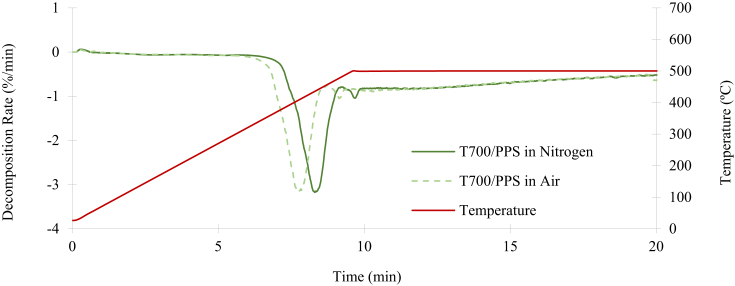
Decomposition rate of T700/PPS in nitrogen and air.

Fig. 9 illustrates the TGA of T700/PPS in nitrogen and air. It can be observed that oxidation occurs following a short exposure to the temperature of 500°C in air, leading to the mass decomposition of the entire system. This result is comparable to the T700/PA6/6 system and it is denoted by the dashed green line in Fig. 9. However, the temperature of the TGA process had to be increased to 700°C to ensure complete decomposition of all the Kevlar® fibres. This temperature increase was due to the stitching of T700/PPS being Kevlar®, which has a decomposition of over 500°C. In the same way as the previous experiment, the mass reduction did not stabilise after three hours, indicating that even the fibres are being oxidised. Subsequently, the same procedure was repeated using nitrogen as the purge gas, as soon as the temperature reaches 500°C, the process was set to be isothermal for 90 minutes until the mass decomposition stabilises at 75.4%. At this point, only the T700 carbon and Kevlar® fibres remained. The procedure was repeated at 700°C until the mass reduction converges to 70.6%, indicating the mass percentage of the remaining T700 fibres. The mass fraction of the Kevlar® stitching was calculated following the two-step TGA process of the T700/PPS. The sample's mass degradation rates for the T700/PPS system in shown in Fig. 10. During the testing period of three hours, the peak degradation rate of the T700/PPS is −3.09%/min in nitrogen and −3.13%/min in air.

With regards to the T700/PA6.6, each sample consists of T700 carbon fibre, a thermoplastic polymer and water or moisture or void (since the stitching material for this system is also PA6.6). With respect to T700/PPS, each sample is comprised of T700 carbon fibre, a thermoplastic polymer, water and stitching material, Kevlar®.

The mass recorded by the thermogravimetric analyser was then converted into volume of the constituent materials using their known densities () and the following equation

(1) $$\rho =\frac{m}{V}$$

where ρ is the density, m is the mass and V is the volume.

Therefore, for the T700/PA6.6 system, the total volume

(2) $$100\%\;Volume=V_{f}+V_{p}+V_{w}$$

And for the T700/PPS system, the total volume

(3) $$100\%\;Volume=V_{f}+V_{p}+V_{w}+V_{K}$$

Using the information obtained in Tables 2 and 3, Eqs. (1), (2), and (3), Tables 4 and 5 were produced.

**Table 3:** Details of raw material samples.

No. of Samples	Material	Average Sample Mass (mg)
5	T700/PA6.6	42.0
5	T700/PPS	40.8
3	T700	1.1
3	PA6.6	1.4
3	PPS	1.8
3	Kevlar	1.1

**Table 4:** Density of all constituent materials of the T700/PA6.6 and T700/PPS.

Material	Density, ρ($\mathrm{kg}/m^{3}$)
T700/PA6.6	1.485
T700/PPS	1.553
T700	1.800
PA6.6	1.140
PPS	1.370
Kevlar	1.440

**Table 5:** Volume fractions of the fibre, polymer, water/void and Kevlar® stitching (for the T700/PPS).

Material	Volume			
	Fibre, $V_{f}$(%)	Polymer, $V_{p}$(%)	Water/Void, $V_{w}$(%)	Kevlar®, $V_{K}$(%)
PA6.6(1)	52.6	46.9	0.5	
PA6.6(2)	51.9	47.2	0.9	
PA6.6(3)	52.1	47.4	0.5	
PA6.6(4)	53.4	45.9	0.7	
PA6.6(5)	51.7	47.7	0.6	
**Average (%)**	52.3	47.0	0.6	
**CV (%)**	1.3	1.5	26.1	
PPS(1)	61.1	33.1	1.0	4.8
PPS(2)	61.6	32.4	1.3	4.7
PPS(3)	60.2	31.7	3.0	5.1
PPS(4)	59.8	32.4	2.4	5.4
PPS(5)	61.1	33.4	1.2	4.3
**Average (%)**	60.8	32.6	1.8	4.9
**CV (%)**	1.2	2.0	49.0	8.6

Based on Table 4, the FVFs of the T700/PA6.6 and T700/PPS are 52.3% (CV=1.3%) and 60.8% (CV=1.2%), respectively. There was a considerable volume fraction of the Kevlar® stitching in the T700/PPS, at 4.9% on average CV=8.6%). These findings could potentially influence the behaviour and mechanical properties of the material; therefore, it should be accounted for when performing its mechanical characterisation. The void content of the T700/PA6.6 panels of 0.6% was calculated to be lower than that of the T700/PPS, which is 1.8%. Subsequently, the porosity of the T700/PPS panels was found to be 200% higher than that of the T700/PA6.6. This result confirms that the T700/PPS has a poorer fibre-matrix bond in comparison to the T700/PA6.6 (which will later be discussed following the fractographic analysis of the fibres).

**Fig. 11:**
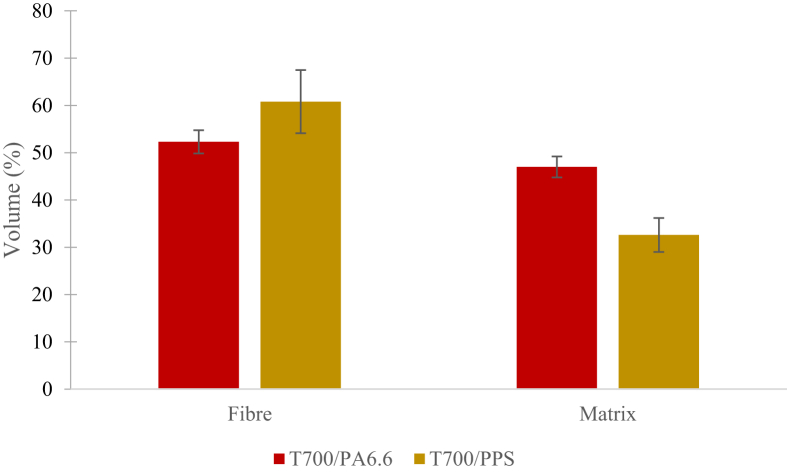
The FVF of both NCF CFRTP systems and the discrepancies between the FVF claimed by the manufacturer and measured using TGA (denoted by the error bars).

The material supplier claimed that both NCF CFRTP prepregs to be approximately 55%. However, using the TGA method, based on Fig. 11, the FVF for the T700/PA6.6 and T700/PPS were found to be 52.3% (discrepancy of ∼5%) and 60.8% (discrepancy of ∼11%), respectively.

In conclusion, it was found that the TGA performed on both materials (T700/PA6.6 and T700/PPS) is an efficient technique for determining the fibre volume of the CFRTP systems. The TGA method produces accurate measurements of the volume fractions of the constituent materials and voids in an NCF composite system as the process can be methodically designed to remove individual component of the material via pyrolysis.

## 6 Analysis

**Fig. 12:**
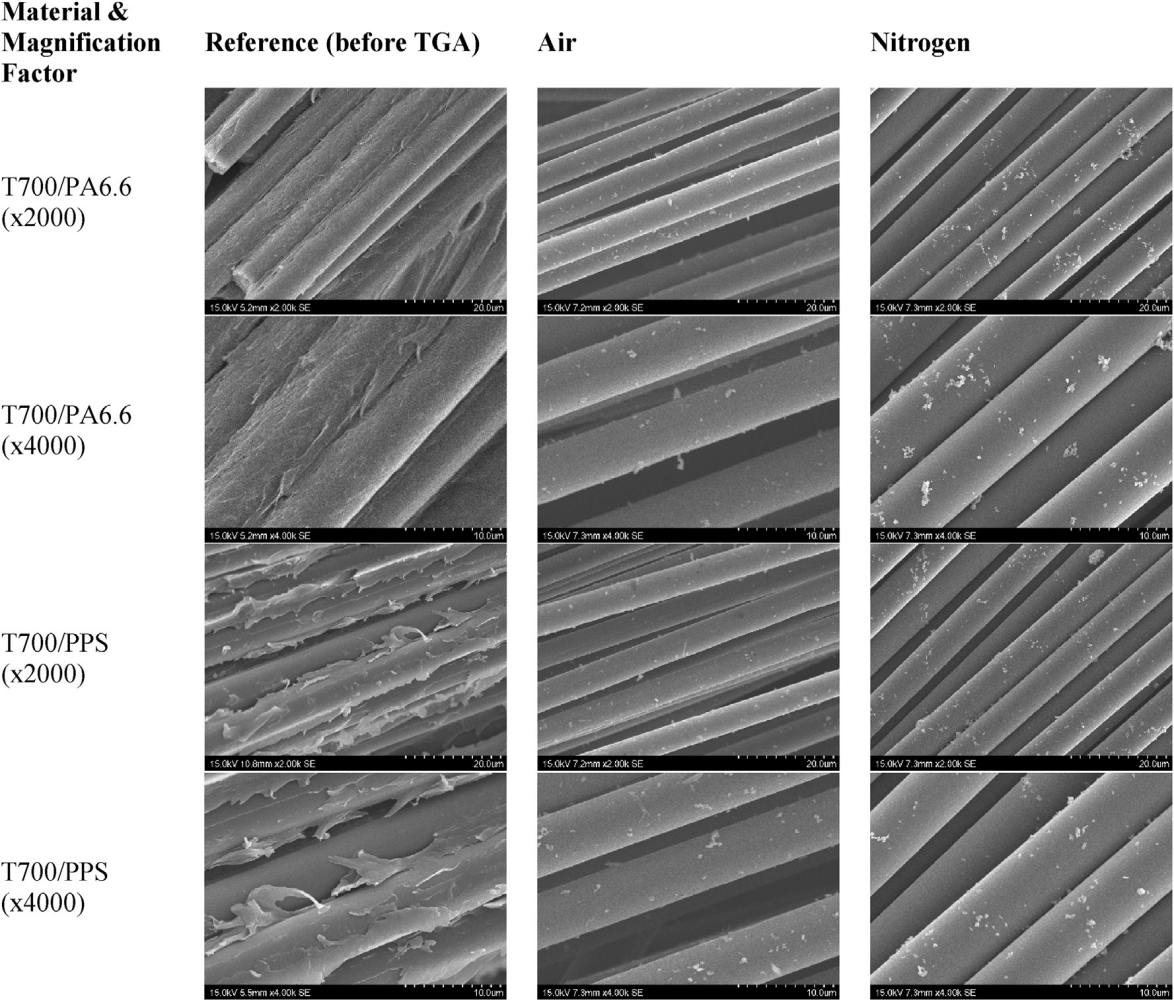
SEM micrographs of the T700/PA6.6 and T700/PPS before and after TGA with different purge gases.

Fig. 12 depicts the SEM micrographs of the fibres before and after TGA. The T700 fibres in the T700/PA6.6 and T700/PPS systems (left column of Fig. 12) before TGA are coated with their respective thermoplastic polymers. The first and second row of Fig. 12 indicate the T700/PA6.6 system after TGA, 500°?? for three. The third and fourth row of Fig. 12 depict the T700/PPS system after TGA, 500°?? for 100 minutes and 700°?? for 80 minutes.

After the TGA was conducted using air as the purge gas, it was observed that for both CFRTP systems, the T700 carbon fibres were partially oxidised. This is indicated by the rough surfaces of the fibres shown in all micrographs found in the second column of Fig. 12. The oxidisation resulted in erosion in the fibres yielding the nonuniform surfaces of the fibres.

In comparison, when the TGA was performed using nitrogen as the purge gas, the surfaces of the T700 remained smooth, where the diameter of the fibres remained uniform across their lengths (as shown in the third column of Fig. 12).

The micrographs in the first column of Fig. 12 (before TGA) also validated that the T700/PPS contains more voids than the T700/PA6.6 system. This is likely due to the microporous nature of the PPS used in the T700/PPS composite system and its relatively poor fibre-matrix bonding.

## 7 Conclusions

Following the TGA, it was calculated that average FVF of the T700/PA6.6 is 52.3% (CV=1.3%) and its void content is 0.6% (CV=26.1%). With respect to the T700/PPS system, its average FVF is 60.8% (CV=1.2%), void content is 1.8% (CV=49.0%) and the percentage of Kevlar® stitching content is 4.9% (CV=8.6%). The SEM micrographs also indicated that the fibre-matrix bonding of the T700/PPS is inferior to that of the T700/PA6.6.

It can be concluded that the TGA performed on the both T700/PA6.6 and T700/PPS is an effective method for determining the fibre volume fraction of the CFRTP systems. The TGA method also allows for very accurate measurements of the volume fractions of the constituent materials and voids in an NCF composite system which comprised of stitching made of a third material as the process can be methodically designed to remove individual component of the material via pyrolysis. Thus, TGA provides a viable alternative method to the more labour-intense methods in measuring the FVF of CFRTP such as acid digestion, burn off and optical measurement.

Future research could potentially investigate on whether the TGA technique FVF measurement could be applied to short fibre reinforced composites (such as chopped fibre composites) or hybridised CFRTP.

## Declarations

### Author contribution statement

Muhammad A. A. Mohsin: Conceived and designed the experiments; Performed the experiments; Analyzed and interpreted the data; Contributed reagents, materials, analysis tools or data; Wrote the paper.

Lorenzo Iannucci: Analyzed and interpreted the data; Contributed reagents, materials, analysis tools or data.

E. S. Greenhalgh: Conceived and designed the experiments; Analyzed and interpreted the data; Contributed reagents, materials, analysis tools or data.

### Funding statement

This work was supported by the Malaysian Government and partners of DATACOMP and THERMOCOMP project.

### Competing interest statement

The authors declare no conflict of interest.

### Additional information

No additional information is available for this paper.
